# Supplementary figures and images for: Integrin β1/FAK/SRC signal pathway is involved in autism spectrum disorder in Tspan7 knockout rats
Source: Life Sci Alliance. 2022 Dec 20;6(3):e202201616. doi: 10.26508/lsa.202201616 (PMC9768919; doi:10.26508/lsa.202201616)

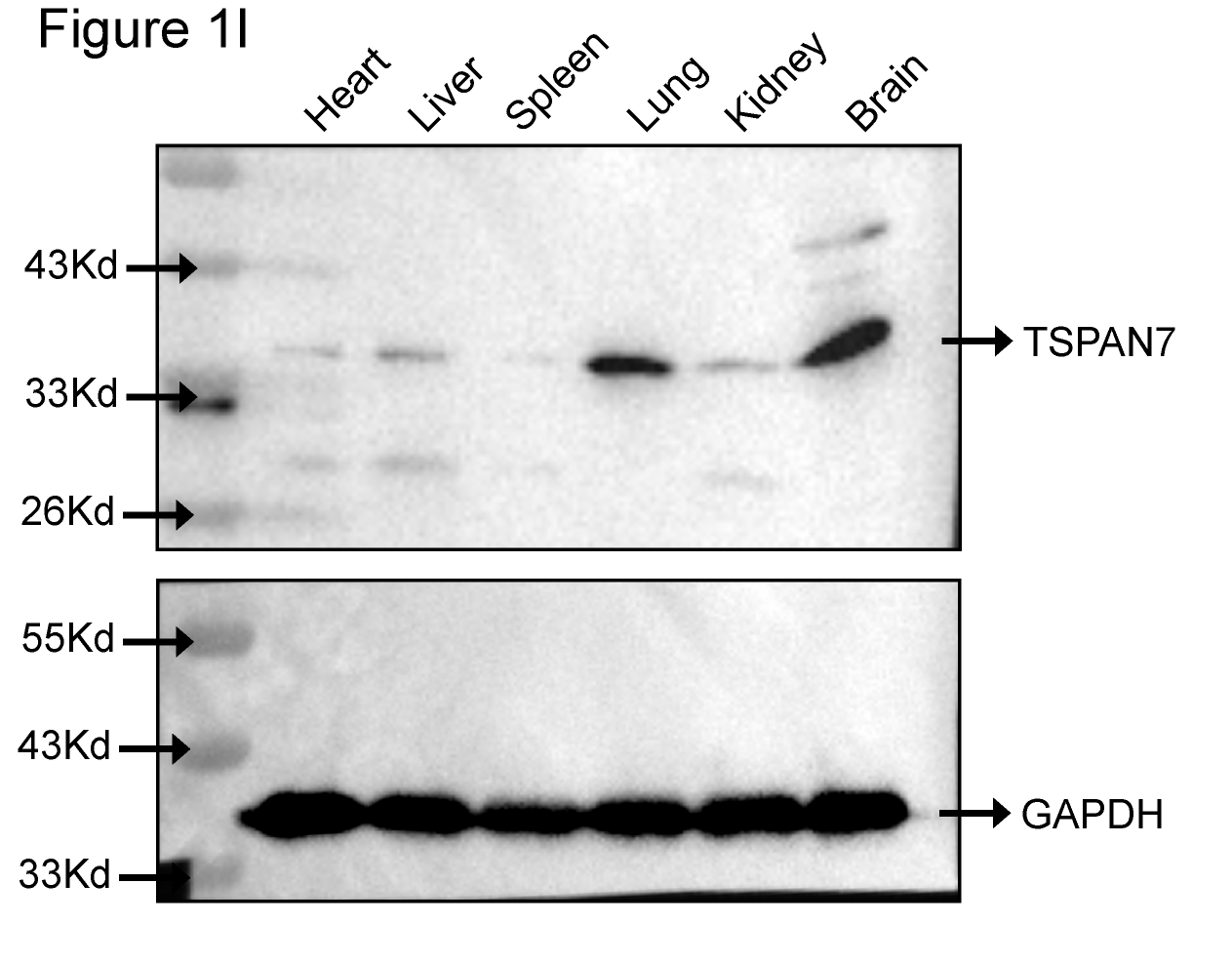

Supplement: Supplementary file 2 [file LSA-2022-01616_SdataF1.2.tif]

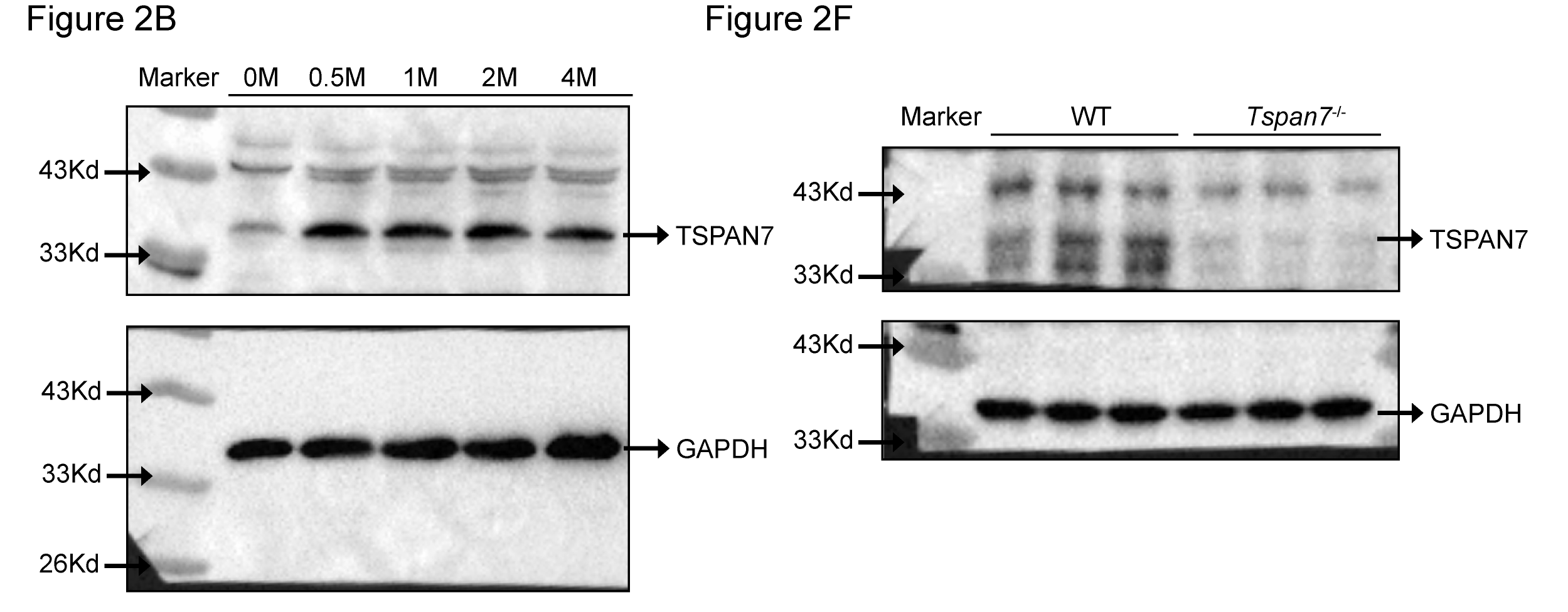

Supplement: Supplementary file 3 [file LSA-2022-01616_SdataF2.1.tif]

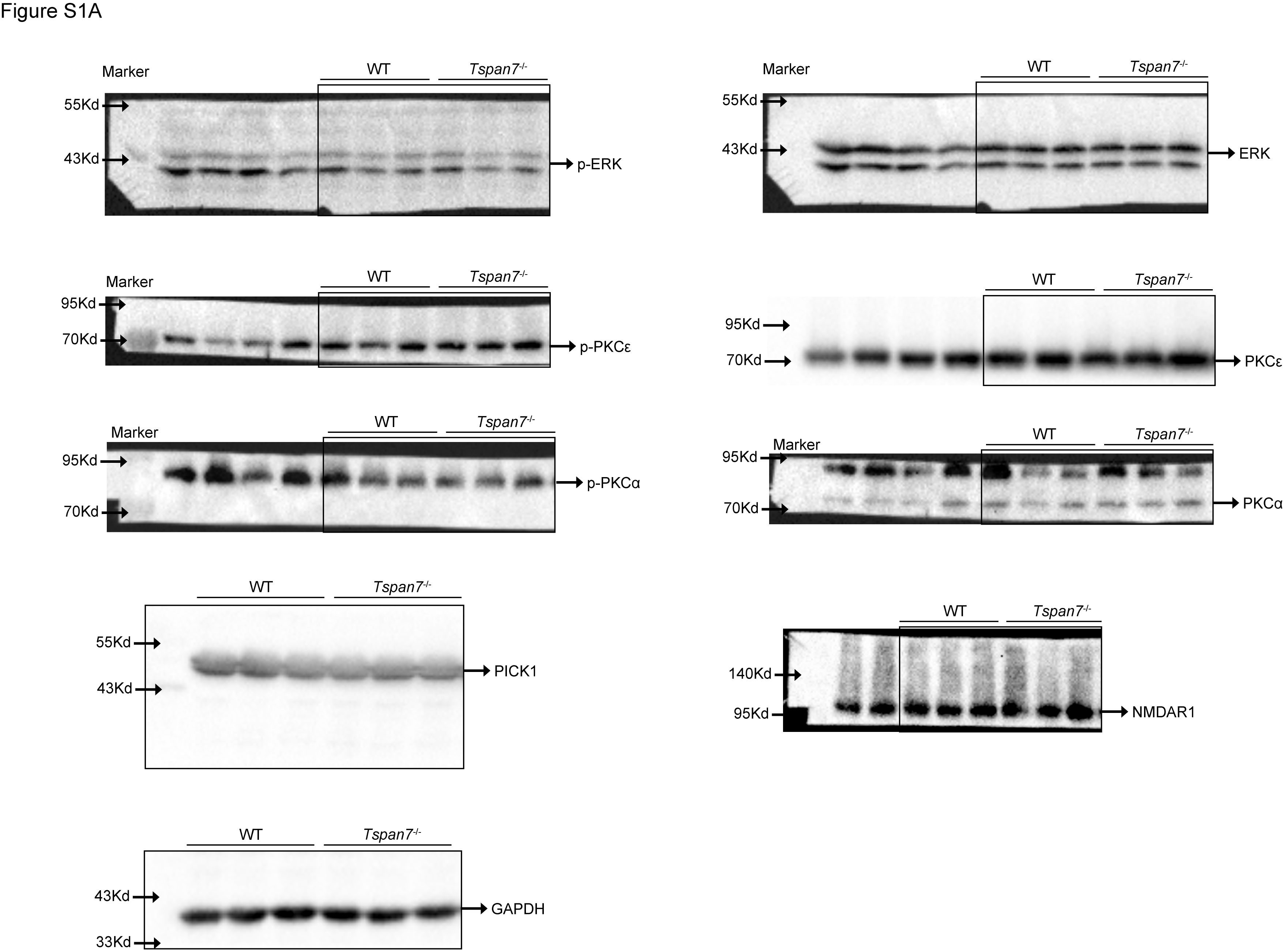

Supplement: Supplementary file 5 [file LSA-2022-01616_SdataFS1.1.tif]

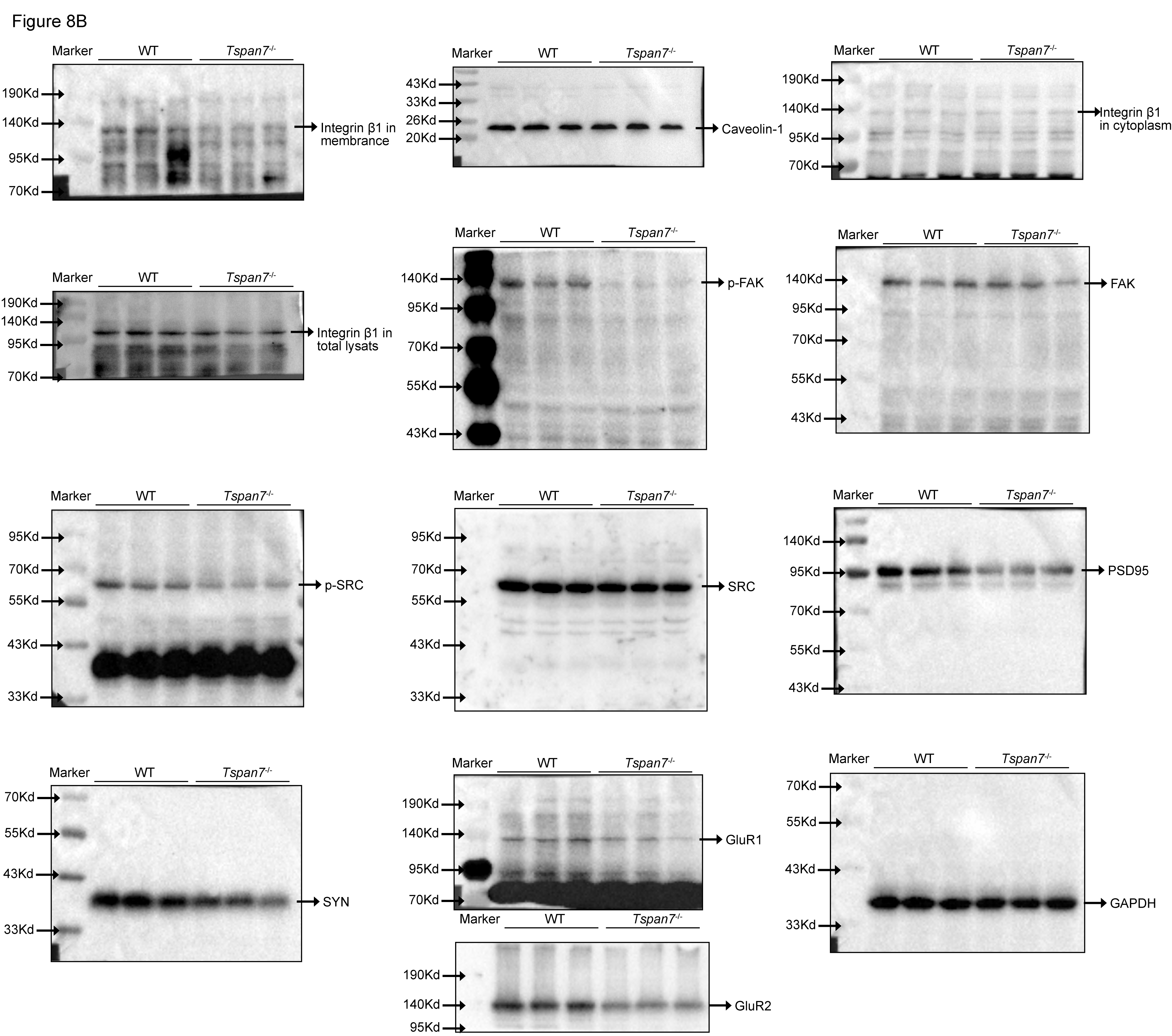

Supplement: Supplementary file 12 [file LSA-2022-01616_SdataF8.1.tif]

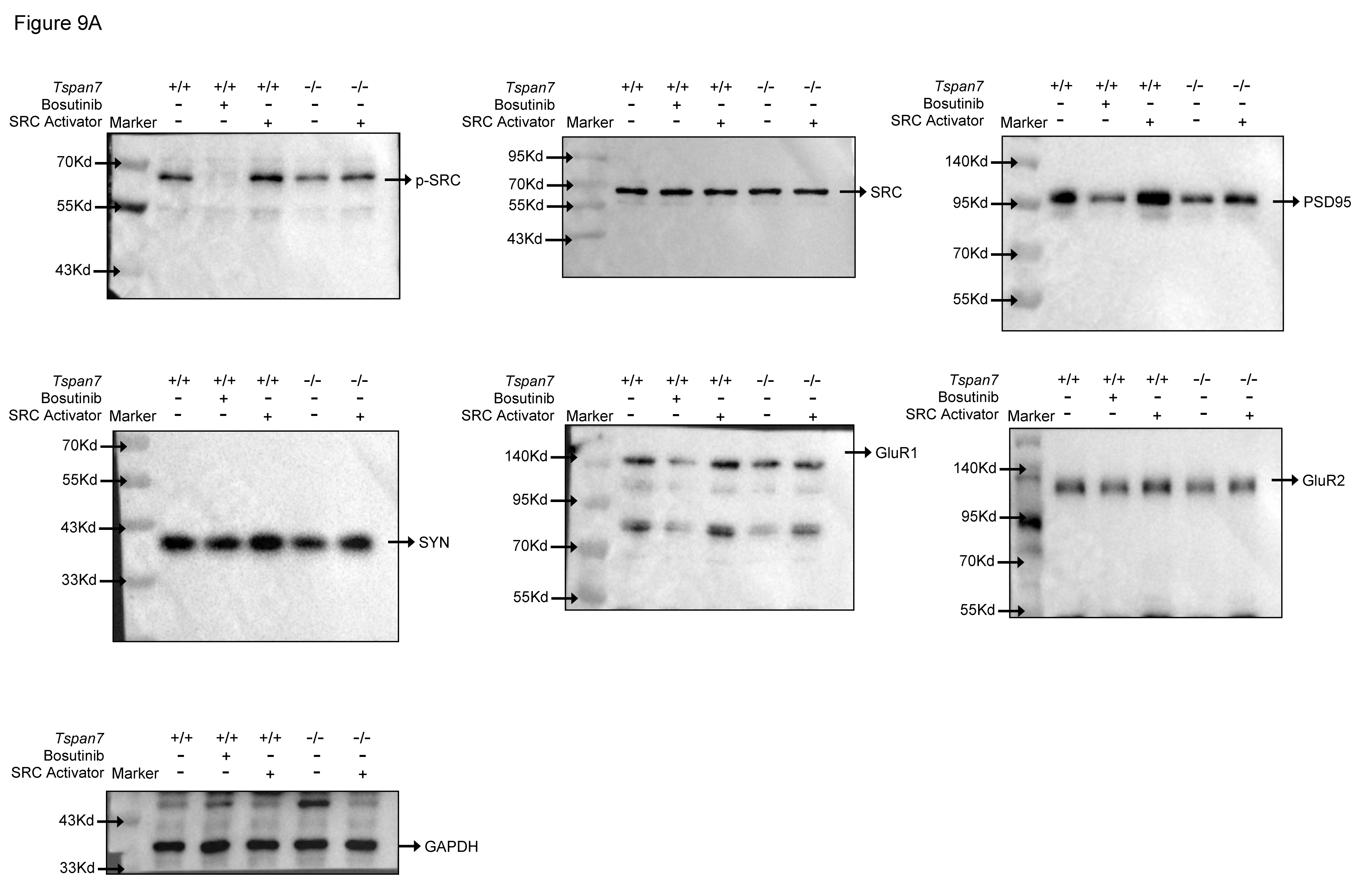

Supplement: Supplementary file 14 [file LSA-2022-01616_SdataF9.1.tif]
